# Parentage Atlas of Italian Grapevine Varieties as Inferred From SNP Genotyping

**DOI:** 10.3389/fpls.2020.605934

**Published:** 2021-01-15

**Authors:** Claudio D’Onofrio, Giorgio Tumino, Massimo Gardiman, Manna Crespan, Cristina Bignami, Laura de Palma, Maria Gabriella Barbagallo, Massimo Muganu, Caterina Morcia, Vittorino Novello, Anna Schneider, Valeria Terzi

**Affiliations:** ^1^Department of Agriculture, Food and Environment, University of Pisa, Pisa, Italy; ^2^CREA Research Centre for Genomics and Bioinformatics, Fiorenzuola d’Arda, Italy; ^3^CREA Research Centre for Viticulture and Enology, Conegliano, Italy; ^4^Department of Life Sciences, University of Modena and Reggio Emilia, Modena, Italy; ^5^Department of Sciences of Agriculture, Food, Natural Resources and Engineering, University of Foggia, Foggia, Italy; ^6^Department of Agricultural, Food and Forest Sciences, Università degli Studi di Palermo, Palermo, Italy; ^7^Department of Agriculture and Forest Sciences, University of Tuscia, Viterbo, Italy; ^8^Department of Agriculture, Forestry and Food Sciences, University of Turin, Turin, Italy; ^9^Research Council of Italy, Institute for Sustainable Plant Protection, Turin, Italy

**Keywords:** cultivar geographic areas, Italian germplasm, Italian founder varieties, parent-offspring relationships, pedigree, second-degree relationships, single nucleotide polymorphism, *Vitis vinifera*

## Abstract

The Italian grape germplasm is characterized by a high level of richness in terms of varieties number, with nearly 600 wine grape varieties listed in the Italian National Register of Grapevine Varieties and with a plethora of autochthonous grapes. In the present study an extended SNP genotyping has been carried out on Italian germplasm of cultivated *Vitis vinifera* subsp. *sativa* and *Vitis* hybrids. Several hundred Italian varieties maintained in the repositories of scientific Institutions and about one thousand additional varieties derived from previous studies on European, Southern Italy, *Magna Graecia* and Georgian germplasm were considered. The large genotyping data obtained were used to check the presence of homonyms and synonyms, determine parental relationships, and identify the main ancestors of traditional Italian cultivars and closely-related accessions. The parentage among a set of 1,232 unique varieties has been assessed. A total of 92 new parent-offspring (PO) pairs and 14 new PO trios were identified. The resulted parentage network suggested that the traditional Italian grapevine germplasm originates largely from a few central varieties geographically distributed into several areas of genetic influence: “Strinto porcino” and its offspring “Sangiovese”, “Mantonico bianco” and “Aglianico” mainly as founder varieties of South-Western Italy (IT-SW); Italian Adriatic Coast (IT-AC); and Central Italy with most varieties being offsprings of “Visparola”, “Garganega” and “Bombino bianco”; “Termarina (Sciaccarello)” “Orsolina” and “Uva Tosca” as the main varieties of North-Western Italy (IT-NW) and Central Italy. The pedigree reconstruction by full-sib and second-degree relationships highlighted the key role of some cultivars, and, in particular, the centrality of “Visparola” in the origin of Italian germplasm appeared clear. An hypothetical migration of this variety within the Italian Peninsula from South to North along the eastern side, as well as of “Sangiovese” from South to Central Italy along the Western side might be supposed. Moreover, it was also highlighted that, among the main founders of muscat varieties, “Moscato bianco” and “Zibibbo (Muscat of Alexandria)” have spread over the whole Italy, with a high contribution by the former to germplasm of the North-Western of the peninsula.

## Introduction

The cultivated grapevine, *Vitis vinifera* L. subsp. *sativa* (DC.) Hegi, is one of the major horticultural species worldwide (Vivier, 2002), firstly domesticated about 8–10,000 years ago from its wild relative, the dioecious *V. vinifera* L. subsp. *sylvestris* (Gmel.) Hegi, in the region that extends from the South Caucasus to the Fertile Crescent to Central Asian countries. From there viticulture spread across Europe and North Africa (Levadoux, 1956; Zohary, 1996; McGovern, 2004; This et al., 2006; Forni, 2012; Bacilieri et al., 2013).

The shift from dioecy to hermaphroditism was a milestone in *V. vinifera sylvestris* domestication. After this crucial step, the genetic diversity of domesticated, hermaphrodite *V. vinifera sativa* was historically modeled by three main forces: sexual propagation (reproduction), vegetative propagation (multiplication), and somatic mutations. In addition to open pollination, self-fertilization can also create novel allelic combinations, as the grapevine is a highly heterozygous species. Hermaphrodite grapevine genotypes can theoretically be selfed until homozygosity, however, such progenies are disadvantaged because of the effect of deleterious alleles exposed in the homozygous status and inbreeding depression (Zhou et al., 2019). Consequently, most cultivated *V. vinifera sativa* varieties are highly heterozygous, resulting from combined spontaneous or controlled hybridization and the selection of somatic mutations.

The ancient, traditional varieties are the result of spontaneous crosses, involving the planting of the recombinant seeds and/or somatic mutations that were selected and propagated centuries ago. Reliable documentation on their origins is lacking. On the other hand, modern varieties, dating from 1,800 onward, resulted from controlled crosses and selection aimed at improving different viticultural traits such as pathogen tolerance, ripening time, crop yield, berry size, color, and flavor.

The selection of desirable traits is a long process in grapevines. Once identified, the suitable varieties are multiplied by cuttings to ensure genetic stability. The wide use of the most interesting parents likely favored the emergence of groups of related cultivars, as observed in the most traditional viticultural regions (Myles et al., 2011; Lacombe et al., 2013; Zinelabidine et al., 2015). However, clonal variants can rapidly appear in such asexually generated plants and can be transmitted by sexual propagation depending on the specific mutated cell layer (reviewed by This et al., 2006). In addition, the co-presence of wild and domesticated plants along the migratory routes gave rise to gene flows between the subspecies, and further shaped the cultivated genetic pool (Grassi et al., 2003; Arroyo-García et al., 2006; Di Vecchi-Staraz et al., 2008; Riaz et al., 2018; D’Onofrio, 2020).

The cultivated grapevine germplasm has therefore been modeled by such genetic processes, and human intervention has expanded the cultivation area of this perennial crop for millennia, increasing the level of genetic diversity. On the other hand, grapevine diversity was drastically reduced by the pathogen pressure: a well-known example is the effects of mildew and phylloxera, responsible for the destruction of many European vineyards in the XX century.

The grapevine diversity and the relationships among accessions have been evaluated based on several descriptors, starting with morpho-physiological ones. Cultivated grapevine varieties were first classified on a large scale by Negrul (1946) who considered mainly morphological traits and geographical information, and suggested the existence of three eco-geographic groups named proles: *occidentalis*, *pontica*, and *orientalis*. The proles *orientalis* includes grapevine varieties from Central Asia and the Caucasus, the proles *pontica* comprise the varieties from Georgia to the Balkans and most of Italy, while the proles *occidentalis* include the varieties from Western Europe.

Recent studies on the analysis of the polymorphism of DNA molecular markers, and particularly of microsatellite SSRs and more recently of single nucleotide polymorphisms (SNPs), confirmed the classification of Negrul and have also provided evidence that the cultivated compartment of *V. vinifera* is genetically structured (Arroyo-García et al., 2006; Myles et al., 2011; Bacilieri et al., 2013; Laucou et al., 2018; De Lorenzis et al., 2019).

Parentage studies on DNA analysis have also been used to evaluate the importance of sexual crosses and somatic mutations in the generation of traditional grapevine varieties, to identify successful varieties in creating progeny, and to clarify homonyms and synonyms. Since the early 1990s, microsatellite markers have been used as the election markers to identify suspected synonyms and homonyms based on ampelographic studies or those still unknown, and to characterize germplasm collections. Several open access microsatellite databases have thus been implemented.

More recently, SNP arrays have been developed from grapevine genome sequencing projects (Jaillon et al., 2007; Canaguier et al., 2017) and applied for high throughput genotyping (Laucou et al., 2018). SNP analyses based on hundreds or thousands of markers have led to a higher logarithm of the odds (LOD) scores than microsatellite SSRs. Consequently, the capacity to distinguish full-sibling vs. second-degree (SD) relationships has been increased as has the power of parentage studies (Cabezas et al., 2011; Laucou et al., 2018).

With a mean wine production of approximately 50 Mio hL/year, Italy covers about 20% of the world production (OIV, 2019). The Italian production is characterized by a high level of diversification in terms of grapevine varieties and, to date, nearly 600 wine grape varieties are listed in the Italian National Register of Grapevine Varieties. SSR markers have been widely applied for the identification of synonyms and homonyms, and for the parentage analysis of grapevine varieties locally or widely cultivated in Italy. Some of these studies are focused on the reconstruction of the pedigree and the parentage of specific varieties, e.g., Sangiovese (Di Vecchi Staraz et al., 2007), or a family of varieties, e.g., Muscats (Crespan and Milani, 2001), while other studies focus on local, or national SSR-genotyped collections (Costantini et al., 2005; Cipriani et al., 2010; Bacilieri et al., 2013; Emanuelli et al., 2013; Lacombe et al., 2013; De Lorenzis et al., 2014; Štajner et al., 2014; D’Onofrio et al., 2016).

The *Italian Vitis Database* (http://www.vitisdb.it/; D’Onofrio and Scalabrelli, 2010) and the *Registro Nazionale delle Varietà di Vite*^1^ collect ampelographic and ampelometric descriptions, and biochemical and microsatellite profiles of about 800 grapevine varieties cultivated in the Italian Peninsula.

To date, SNPs markers have been used to evaluate parentage in Italian “thematic” germplasm collections, e.g., Muscats grapevine varieties (Ruffa et al., 2016), varieties from Sicily and Calabria (Mercati et al., 2016; Sunseri et al., 2018; De Lorenzis et al., 2019), or from the whole peninsula (Laucou et al., 2018).

All these studies have identified many synonyms, homonyms, and parentage relations among Italian varieties, and provide evidence on the complex and admixed structure of Italian germplasm. However, only a fraction of the Italian germplasm has been studied. A wider-scale evaluation of the parentage among Italian varieties, as well as of their relationships with European and international varieties, is still lacking.

To fill this gap, the present study analyzed the parentage among several hundred accessions of *V. vinifera* subsp. *sativa* and *Vitis* hybrids based on SNP genotyping data collected from Italian germplasm maintained in the repositories of scientific Institutions and about one thousand additional varieties derived from previous studies on European, Southern Italy, *Magna Graecia* and Georgian germplasm. The large genotyping data obtained were used to (i) check the presence of homonyms and synonyms, (ii) determine parental relationships, and (iii) identify the main ancestors of traditional Italian cultivars and closely-related accessions.

## Materials and Methods

### Plant Material and DNA Preparation

A total of 615 grapevine accessions were sampled from the collections of the CREA Research Centre for Viticulture and Enology in Conegliano (343), and from local collections in Piedmont (79 accessions) managed by the Italian National Research Council and the University of Turin; in Emilia-Romagna and Lazio (54 accessions) managed by the University of Modena and Reggio Emilia and the University of Tuscia; in Tuscany (90 accessions) managed by the University of Pisa; in Apulia (27 accessions) managed by the University of Foggia and in Calabria and Sicily (22 accessions) managed by the University of Palermo. The list of sampled accessions is reported in Supplementary File 1.

For DNA extraction young leaf tissues were sampled and genomic DNA was extracted using a CTAB-based buffer followed by chloroform extraction. The DNA samples were checked for quality and quantity.

### Genotypic Data

The accession panel was genotyped using 18,775 SNP markers from the Infinium 18K Grape Array (Illumina Inc., San Diego, CA, United States). Two hundred nanograms of genomic DNA were delivered to TraitGenetics GmbH (Gatersleben, Germany) and were used as a template for array hybridization reactions, following the manufacturer’s instructions (Illumina Inc.). Array signals were converted into discrete genotypes using GenomeStudio (Illumina Inc., San Diego, CA, United States). They were then merged with publicly-available SNP datasets (De Lorenzis et al., 2015, 2019; Laucou et al., 2018) which include 1042 varieties from the main historical grape growing areas.

A missingness rate > 1% and a minor allele frequency (MAF) < 5% were allowed for markers. Subsequently, individual missingness and heterozygosity were calculated and individuals with more than 5% of missing values were excluded. Duplicated accessions were detected by calculating the pairwise percentage of mismatch between individuals, to discuss homonyms, synonyms and possible accession misnaming.

### Parentage and Relatedness

Pedigree reconstruction of grapevine accessions included in the panel focused first on the detection of parent-offspring (PO) relationships. Secondly, full-sibs (FS) and SD relationships were investigated, to extend the reconstructed pedigree toward ancestors as much as possible.

Single PO relationships (duos) were detected based on two metrics: (i) count of Mendelian errors; (ii) identity by descent (IBD) coefficients (k0, k1, and k2) estimated by PLINK’s method of moment implemented in the R package SNPRelate (Zheng et al., 2012).

Stricter filters were applied to the dataset before parentage analysis. Individuals with a missingness > 1%, heterozygosity < 25% and > 45% were removed. In addition, for estimation of IBD coefficients, SNPs were pruned based on linkage disequilibrium (LD) greater than 0.8. POs were identified by examining the distribution of Mendelian errors and the k0 coefficient across the whole population. The two distributions were bimodal, with a clear gap around k0 = 0.03 between putatively true POs and false POs. All the relationships with k0 < 0.03 were considered PO duos. Subsequently, Mendelian errors were counted for all the possible trios (two parents and one offspring) emerging from the duos identified in the previous step.

Full-sib relationships were detected based on the IBD coefficients (k0, k1, and k2). Since a clear cutoff between FS and half-sibs was not evident, a conservative threshold (k2 > 0.3) was used to identify only those full-sib relationships that were well supported by the data.

Second-degree relationships have been used to extend the reconstruction of a pedigree toward progenitors when a lineage is missing a parent and direct relationships cannot be used. This approach is more powerful by focusing on the genotypes of a known PO pair (i.e., a trio with a missing parent) to identify SD relatives of the offspring (i.e., parents, progenies or full-siblings of the missing parent). In likelihood-based methods for pedigree reconstruction, such as the one implemented in the software COLONY (Jones and Wang, 2009) or SEQUOIA (Huisman, 2017), this problem is usually referred to as (half-)sibship clustering, since the aim is to identify half-sibs of the focal individual (the offspring). In this study, we used a simplified method presented by van de Weg et al. (2018) that is based on the same approach, while it is not using computationally intensive likelihood calculations.

Briefly, given a known PO pair and a subset of markers with AA (for the putative parent) and AB (for the putative offspring), the number of BB and AB genotypes is counted for each possible related individual. Since for those markers the known (putative) parent is AA, the B allele must have been inherited from the missing parent lineage. Therefore, individuals with a high frequency of B alleles for those markers are likely to be SD relatives of the offspring (i.e., half-sibs, grandparents or avuncular relationships), belonging to the pedigree lineage of the non-genotyped missing parent. However, since the direction of a PO relationship is usually unknown, for each PO pair, the method for SD identification is performed twice, once for each PO direction. Consequently, evidence of the presence of SDs can also provide an indication of the most probable direction of a PO relationship. When a set of possible SDs is identified, Mendelian errors can be used to test the presence of a couple of grandparents (i.e., parents of the missing parent).

For the founders of the Italian germplasm, when sibship clustering could not be informative (founders do not have parental genotypes to be used as condition), IBD coefficients have been used to further explore their relationships. Although IBD coefficients are not accurate enough to clearly define SD or higher degree relationships, they could still indicate groups of varieties that are more closely related. However, differently from SD relationships identified by sibship clustering, relatives derived by IBD coefficients could also include descendants, that are less relevant to investigate the origins of a variety.

Except for the estimation of IBD coefficients, all the other parentage and pedigree reconstruction analyses were conducted using a set of custom-made R scripts. Networks of relationships were plotted using the R package “network” (Butts, 2015) or manually.

## Results

A large panel of 615 grape accessions (including mainly Italian varieties) was genotyped in this study by using a 18K SNP array. Data were merged with three public datasets, containing SNP data of 1,042 genotypes from the main historical viticultural areas, to study genetic relationships with Italian grape material.

Heterozygosity and missingness per individual (Supplementary File 3) were calculated based on a set of 6,770 high-quality SNPs (Supplementary File 2) remaining after filters for missingness and MAF filters were applied. Individuals with more than 5% missingness (23 individuals) showed a higher heterozygosity, probably indicating a higher rate of markers with unclear clustering of array hybridization signals due to low-quality DNA samples. Excluding samples with more than 5% missingness, mean heterozygosity was 33%, in agreement with previous studies based on SNP data (Laucou et al., 2018). It should be noted that this represents a large underestimation of the actual heterozygosity of individuals, since there is a high chance that SNP alleles are identical by state (IBS), but not identical by descent (IBD).

### Duplicated Accessions, Homonyms and Synonyms

The genetic dissimilarity between accessions was studied by calculating the pairwise percentage of mismatch in the 6,770 high-quality SNPs. A clear two-mode distribution was observed, with very similar accessions having a maximum mismatch of 0.12%, while non-identical accessions showed a minimum mismatch of 12.6%. Accessions with less than 0.12% mismatch were considered identical. The comparison of the somatic variants suggested that the somatic mutations cannot explain any of observed mismatch fraction of the 6,770 high-quality SNPs considered, and that all the fraction of mismatches less than 0.12% is due to genotyping errors. Accessions were grouped into clusters of identical individuals to study synonyms, homonyms, and accession misnaming. Approximately 25% of the profiles were duplicates, due to an overlap between datasets, synonyms, clones, or accession misnaming. A total of 1,232 unique varieties was identified: 408 from this study and 824 from public datasets (Supplementary Files 1, 2).

Among the set of 615 accessions genotyped in this study, 82 (13.3%) were synonyms, 12 (2.0%) somatic variants, 17 (2.8%) were wrong denominations, and 5 (0.8%) were misnomers. We considered as “wrong denominations” incorrect local names of known varieties and that we marked as “false”. On the other hand, we considered errors in grapevine collections as “misnomers”. Additional 56 synonyms were found after comparison with the public datasets (Supplementary File 1).

### Parentage Analysis

First-degree relationships were investigated using Mendelian errors and estimations of IBD coefficients (k0, k1, and k2). All 829 PO relationships identified by IBD coefficients (k0 < 0.03) were confirmed by Mendelian errors (Supplementary File 4). This set of POs involved 767 varieties (62.3%), while no direct relationships were found for the remaining 465 (37.7%) varieties in this panel (Supplementary File 5). A total of 92 duos was identified for the first time in this study while 2 pairs invalidated previous findings (Table 1).

**TABLE 1:** Parent-offspring pairs (POs) reported in this study for the first time.

Name variety 1	Name variety 2	Name variety 1	Name variety 2
Abello	Visparola	Grechetto (di Orvieto)	Pignoletto
Achladi (Pergolese di Tivoli)	Invernenga	Greco nero (di Verbicaro)	Mantonico bianco
Aglianico	Austegna	Guarnaccia (bianca)	Guarnaccino
Aglianico	Nera del Cilento	Italia	Pomposa MO
Albana	Trebbianina	Kakotryghis	Kosinjot
Albana del paniere	Termarina (Sciaccarello)	Kosinjot	Zagarese
Albanello	Vilana	Lambrusco d’German	Orsolina
Aleatico	Lacrima (di Morro d’Alba)	Maiolica	Verdone nero
Alicante (false)	Lucignola	Maiolica	Negro amaro
Alionza	Garganega	Maiolica	San Pietro
Ansonica	Mantonico bianco	Maiolica	Visparola
Aramon	Vigne sauvage faux Mouchouses 1	Malvasia bianca	Termarina (Sciaccarello)
Arilla	Biancolella	Malvasia bianca lunga	Prunesta (false)
Arsilico	Pampanaro	Mammolo clone I-MAM-PA-1	Termarina (Sciaccarello)
Arsilico	Rosso antico	Mantonico bianco	Truccanosa
Arsilico	Maiolica	Molinara	Vulpea
Assoued kere	Grillo (false)	Moscato d’Adda	Terzi 100.31
Balzamino	Gambugliana	Mostosa	Sanguinella
Barsaglina	Visparola	Neretto di Marengo	Verdea noir
Bazaleturi	Krakhouna	Nero di Gonzaga	Turchetta
Bazaleturi	Muradouli	Occhi di Lepre	Sangiovese
Bazaleturi	Tsitska	Orpicchio	Visparola
Bazaleturi	Vertkvitchalis tetri	Orsolina	Uva d’oro bianca
Bellone	Pampanaro	Orsolina	Pattaresca
Blatterle	Schiava gentile	Pate noir	Raisaine
Bombino bianco	Palomba (Uva Carrieri)	Pecorello (falso)	Visparola
Bombino bianco	Uva della macchia	Ragusano	Visparola
Boschera	Vulpea	Ramishvili 07	Tita kartlis
Bracciola nera (false)	Termarina (Sciaccarello)	Rossara toscana	Termarina (Sciaccarello)
Burdin 7419	Gamay	Ruggine	Schiava (Rossara)
Canina nera	Pelagos	Tognona	Verdicchio bianco
Cannonau (Garnacha tinta)	Galibert treblanc	Tosca bianca	Uva Tosca
Capretta	Maturano	Ugillina	Verdicchio bianco
Carraresa	Gargola	Uva Tosca	Vintaiu
Carricante	Maria	Uva Tosca	Vite di Roteglia
Casavecchia	Guarnaccia (false)	Vernaccia di Oristano (Spergola)	Vespaiola
Centesimino	Moscato violetto (Muscat rouge de Madère)	Vernaccina	Visparola
Centesimino	Sangiovese	Zibibbo (Moscato d’Alessandria)	Bianca di Bellosguardo
Cinsaut	Verdelet	Zibibbo (Moscato d’Alessandria)	Signurina
Corinto (falso)	Minnella nera	1) Carmenère	Fertilia
Cornacchia	Lambrusco Grasparossa	1) Couderc 25 (questionable)	Trebbiano toscano
Cornichon blanc	Corniola (false)	2) Barbera	Ervi
Corniola (false)	Kontegalo	2) Bacchus weiss	Geilweilerhof GA 48-12
Covra	Orsolina	2) Cabernet sauvignon	San Lorenzo
Dolciame	Garganega	2) Celtica	Chardonnay
Famoso	Termarina (Sciaccarello)	2) Celtica	Riesling italico
Filucca	Orsolina	2) Croatina	Ervi
Folha de figueira	Uva del Fantini	2) Furmint	Vega
Gabekhouri tsiteli	Mtsklarta	2) Incrocio Bruni 624	Picolit
Galibert Coulondre 15–2	Zibibbo (Moscato d’Alessandria)	2) Muscat de St. Christol (Galibert 255-43)	Muscat reine des vignes
Gradino	Uva Tosca	2) San Lorenzo	Trebbiano toscano

*1) Parent-offspring pairs identified in this study invalidating previous data.2) Parent-offspring pairs identified in this study confirming previous data.(false): wrong denominations (incorrect local names).*

Full parentage (trio) was investigated by counting Mendelian errors for all the possible trios arising from the detected POs. A high discrimination power was observed in the detection of true trios, mainly due to the high number of SNPs and the relatively low genotyping error rate (Supplementary File 6). The maximum number of Mendelian errors observed for the true trios was four, while the minimum number observed for false trios was 115. A total of 189 trios was detected (Supplementary File 7); 14 of which were first identified in this study (Table 2). It is noteworthy that, with the approach used, most of the kinship relationships present in modern grape varieties, included in our set and deriving from controlled crosses declared in pedigree, are confirmed (as is the cases of “Celtica”, “Ervi”, “San Lorenzo”, “Caprugnone”, “Geilweilerhof GA 48 12” and “Incrocio Bruni 624”).

**TABLE 2:** Parents-offspring trios reported in this study for the first time.

Offspring	Parent 1	Parent 2
Alicante (false)	Cannonau (Garnacha tinta)	Lucignola
Casavecchia	Guarnaccia (false)	Malvasia bianca di Candia
Centesimino	Moscato violetto (Muscat rouge de Madère)	Sangiovese
Giosana	Strinto porcino	Visparola
Grilla	Strinto porcino	Visparola
Iusana	Strinto porcino	Visparola
Ladikino	Albanello	Assouad karech
Lievuso	Sangiovese	Mantonico bianco
Malvasia nera di Basilicata	Somarello	Visparola
Occhi di Lepre	Mantonico bianco	Sangiovese
Sanguinella	Bombino bianco	Mostosa
Signurina	Sultanina	Zibibbo (Moscato d’Alessandria)
Zagarese	Kosinjot	Negro amaro
1) Carricante	Strinto porcino	Visparola
2) Caprugnone	Abello	Termarina (Sciaccarello)
2) Celtica	Chardonnay	Riesling italico
2) Ervi	Barbera	Croatina
2) San Lorenzo	Cabernet sauvignon	Trebbiano toscano

*1) Parents-offspring trios identified in this study invalidating previous data.2) Parents-offspring trios identified in this study confirming previous data.(false): wrong denominations (incorrect local names).*

However, examples of discrepancy between genetic data and pedigree have also been highlighted (Tables 1, 2).

**FIGURE 1:**
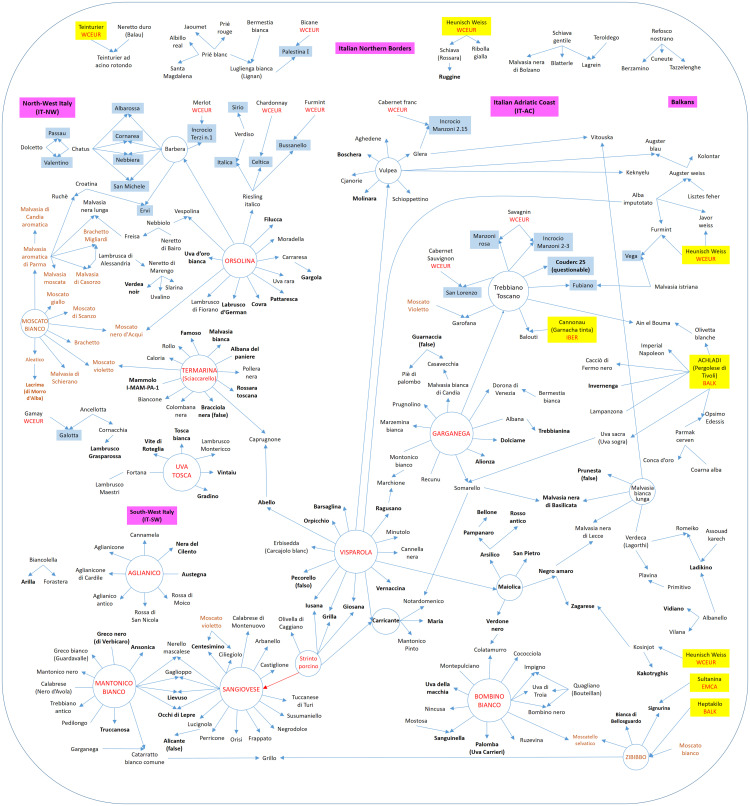
Network of main POs among Italian varieties organized according to their geographic areas. The arrows indicate PO’s direction. Bold names indicate POs reported for the first time in this study. Anthropogenic crosses are highlighted in light-blue. Parents of foreign origin contributing into spontaneous crosses are highlighted in yellow with their alleged geographic groups. Parents from the cluster of Muscat varieties are highlighted in brown. Red connectors refer to still doubtful PO direction. Connectors without arrow refer to PO without direction.

A focus on the Italian varieties including the main connections with foreign varieties is reported in Figure 1. This network of POs contained 16 main non-Muscat genitors with more than five PO relationships with other Italian varieties (black and red varieties circled in Figure 1). “Sangiovese” was the variety with the highest number of POs (17) followed by “Visparola” (with 16 POs), “Mantonico bianco”, “Orsolina”, and “Termarina (Sciaccarello)” (13 POs), “Garganega” and “Bombino bianco” (11 POs), “Trebbiano toscano” and “Vulpea” (9 POs), “Aglianico” (8 POs), “Barbera” (7 POs), “Uva tosca” and “Strinto porcino” (6 POs), “Malvasia bianca lunga”, and “Carricante” (5 POs). In addition, seven small isolated kin groups were identified with 3–4 Italian varieties and 14 solitary PO duos (Supplementary File 8). The Muscat varieties “Moscato bianco” and “Zibibbo (Moscato d’Alessandria)” have also contributed to the diversity of the Italian germplasm (brown varieties circled in Figure 1).

Three new offspring (offs) of “Sangiovese” and of “Mantonico Bianco” were identified in this study (Figure 1). “Visparola” appeared to be related to several Southern and Central Italian varieties. Four trios between “Visparola” and “Strinto porcino” (“Carricante”, “Iusana”, “Giosana” and “Grilla”, the latter being different from the more well-known “Grillo”) and six POs of “Visparola” were reported for the first time in this study (Figure 1 and Supplementary File 5).

“Termarina (Sciaccarello)”, also referred to as a “Mammolo” from Tuscany (Di Vecchi Staraz et al., 2007), was confirmed to be the parent of the “Moscato violetto (Muscat rouge de Madère)” by a cross with “Moscato bianco” as previously reported (Crespan and Milani, 2001; Di Vecchi Staraz et al., 2007), and of “Caprugnone” by a cross with “Abello” (Cipriani et al., 2010). It was also found to be the parent of six new POs from Central and Northern Italy (Figure 1 and Supplementary File 5).

“Orsolina” was found to be a recurrent genitor for many varieties of Central and North-West Italy. Two trios were detected, involving the offspring “Vespolina” (“Nebbiolo” × “Orsolina”) and “Moscato nero di Acqui” (“Moscato bianco” × “Orsolina”). Among others, “Orsolina” was found to be PO-related to the two important wine grapes “Barbera” and “Riesling italico”, confirming prior publication (Figure 1 and Supplementary Files 5, 7).

“Trebbiano toscano” and “Garganega” were confirmed as PO-related. “Alionza” and “Dolciame” were POs of “Garganega” reported for the first time in this study, while all the POs of “Trebbiano toscano” were previously reported (Figure 1 and Supplementary File 5).

“Sanguinella” and “Colatamurro” were identified as two new offspring of “Bombino bianco”, generated from crosses with “Mostosa” and “Verdone nero”, respectively. “Bombino bianco” was also found to be involved in two new POs with Apulian varieties (Figure 1).

Some other new PO relationships were reported for “Aglianico”, “Malvasia bianca lunga”, “Carricante” and “Uva Tosca”, although only “Malvasia nera di Basilicata” was involved in a full parentage trio (Figure 1 and Supplementary Files 5, 7). On the other hand, all the PO relationships of “Barbera” have been reported previously.

“Achladi (Pergolese di Tivoli)”, a Greek variety that also has many relationships with Iberian varieties (Laucou et al., 2018), was confirmed PO with “Uva sacra (Uva sogra)”, “Cacciò di Fermo nero”, while the PO “Invernenga” was reported for the first time in this study (Figure 1 and Supplementary File 5).

### Pedigree Reconstruction

In order to identify the main ancestors of the Italian germplasm, a pedigree reconstruction based on SD relationships has been performed. These results, together with inferred FS relationships and kinship coefficients, were used to provide information on unknown PO directions (Supplementary File 9). More than 10 half-sibship clusters were identified, most of them including several varieties (orange ellipses in Supplementary File 9). “Visparola” was found to be a parent of all its POs, considering its FS relationship with “Avgoustiatis”. This configuration was also supported by several SD relations found when testing “Visparola” as parent (e.g., “Battraube” SD with “Vulpea” or “Baratcsuha szurke” SD with “Alba imputotato”). “Visparola” was also found SD related with “Sangiovese”, when “Sangiovese” was tested as offspring of “Strinto porcino”. This SD relationship suggests that “Strinto porcino” is more likely a parent of “Sangiovese”, rather than an offspring (offs). Several other accessions were identified as SD relatives of “Visparola”: “Rossa di San Nicola”, “Rossa di Moico” (“Aglianico” offs), “Trebbiano antico” (“Mantonico bianco” offs), “Palomba (Uva Carrieri)” (“Bombino bianco” offs), “Biancone”, “Gambugliana” (“Balzamino” offs), “Veltliner gruener” (“Savagnin” offs) and “Zemoasa” (“Heunisch Weiss” offs). In addition, “Visparola” and “Garganega” were found to be grandparents of “Rollo”, which is an offspring of “Termarina (Sciaccarello)”. “Strinto porcino” was found to be a parent of “Olivella di Caggiano”, “Mantonico bianco” being related to the missing parent. “Mantonico bianco” appeared SD related to “Lucignola” and “Aglianico antico”, offspring of “Sangiovese” and “Aglianico”, respectively. “Vernaccina” (offspring of “Visparola”), “Pecorino”, “Pecorello”, and “Cesanese d’Affile” were all SD related, including “Strinto porcino”. Interestingly, IBD coefficients indicated that “Termarina (Sciaccarello)” is closely related to all of them as well (k > 0.13), although it was not possible to classify those relationships.

A large half-sibship cluster included four offspring of “Termarina (Sciaccarello)” (“Caloria”, “Pollera nera”, “Bracciola nera (false)”, and “Rossara toscana”) together with “Vernaccia di Oristano (Spergola)”, “Vespaiola”, “Durella gentile”, and “Albarola”. “Famoso”, another offspring of “Termarina (Sciaccarello)”, was found to be the grandchild of “Alba imputotato” and “Heunisch Weiss”. In addition, “Termarina (Sciacarello)” was SD related to two offspring of “Garganega”: “Alionza” and “Prugnolino”.

“Ansonica” and “Perricone” were both SD related to “Catalanesca”.

Five varieties were found in a sibship cluster with “Uva sacra (Uva sogra)”, when “Uva sacra (Uva sogra)” was tested as offspring of “Achladi (Pergolese di Tivoli)”. This evidence indicated that “Achladi (Pergolese di Tivoli)” is most likely the parent of “Uva sacra” (alias “Uva sogra)”.

Some varieties had several SD relationships with unrelated varieties, or with varieties belonging to different half-sibship clusters. Those varieties appeared particularly interesting, since they likely represent ancient ancestors recurring in several pedigrees. Beyond “Visparola”, that was mentioned above, we could identify “Koevidinka feher”, “Baratcsuha szurke” and “Dureza”. “Baratcsuha szurke” seems to have role in the Balkans, since resulted SD linked to “Alba imputotato”, “Heunisch Weiss” and all the varieties included in the sibship cluster involving “Chasselas”. The sibship cluster of “Chasselas” (including the Italian varieties “Orpicchio” and “Trebbiano romagnolo”) was also related to “Dureza”, that appeared a key ancestor for several other central European varieties. Lastly, “Koevidinka feher” was found SD related to “Trebbiano toscano” (indicating that “Trebbiano toscano” is most likely an offspring of “Garganega”), “Malvasia aromatica di Parma” and the sibship cluster of “Glera”.

In addition, a few grandparent pairs were identified. “Gradino” (offspring of “Uva Tosca”) was found to be grandchild of “Garganega” and “Termarina (Sciaccarello)”. “Sylvaner verde” (“Silvaner gruen”) was an offspring of “Savagnin” and grandchild of “Alba imputotato” and “Heunisch Weiss”.

## Discussion

The present study provided a wide overview of kinship relationships for a large part of the Italian grapevine germplasm. The germplasm we included in the analysis covered most of the Italian wine cultivars registered in *Italian National Catalogue of Grape Varieties* (Italian National Catalogue of Grapevine Varieties) and some other minor varieties of regional interest (*Italian Vitis Database*).

Effort has been addressed to the correct varietal identification of the grapevine materials analyzed. As an example, the identity of “Nerello cappuccio”, an historic black-berry variety typical of the renowned wine producing area around the Etna volcano, in Sicily, is highly questionable. Pastena (1971) asserted there were different varieties under the name of “Nerello cappuccio”. A recent survey in the area showed that about 70% of the vineyards planted under the name of “Nerello Cappuccio” were “Carignan” (“Mazuelo”), and the remaining 30% were a mixture of other known or unknown cultivars (Branzanti et al., 2010). That is why “Nerello cappuccio” in our dataset corresponds to “Carignan”.

The varieties SNP-genotyped in the present work are mostly identified and described within the Italian National Catalogue of Grapevine Varieties (catalogoviti.politicheagricole.it) and/or the *Italian Vitis Database* (vitisdb.it), where also their SSR-based genetic profile is provided. All these information (cultivation area, plant morphology, and possible historic quotations, etc.) embodies a variety background useful for understanding relationships and genetic issues. In fact, minor or even today’s neglected grapes, poorly known, could play a significant role in major variety pedigrees and development, as it will become clearer later.

Kinship analysis appears to be a proper and informative approach to investigate grapevine germplasm founders and their origins. The low number of generations since grapevine domestication (Fournier-Level et al., 2010) and the vegetative propagation of some of the most suitable ancient ancestors to date (Ramos-Madrigal et al., 2019) contribute to an informative reconstruction of grapevine pedigrees back to ancient founders and their origins.

The original routes through which viticulture (and part of ancient grape cultivated germplasm) spread were likely through human colonization from Eastern to Western Mediterranean countries. The North African route to Western Sicily, Sardinia and Southern Spain was led by the Phoenicians. The Balkan route to Italy and Southern Europe was led by the Greeks, followed by the significant contribution of Romans to Central Europe and Northern Balkans. However, the more recent exchange of grapevine material in other directions (even opposite directions) confounded this pattern. Furthermore, varieties could be spread to different regions at the same time (coeval spreading routes), and often the same varieties are present in several areas/countries with different names (Schneider et al., 2014). Reconstruction of grape pedigrees might be hindered by overlapping generations due to the reproduction between coexisting ancient ancestors, local wild grapevines (*V. vinifera* subsp *sylvestris*) and modern varieties. In addition, some ancient varieties may have disappeared or may be neglected, due to their replacement with new ones following epidemics (phylloxera, mildews) or modern production targets. As a consequence of all these aspects, the current pattern of relationships and the area of cultivation appear as the final result of a stratification of events, often indistinguishable on a time scale.

Given this complex scenario, a pedigree reconstruction approach that makes use of SD relationships (in addition to PO relationships) seems particularly effective, considering the need to overcome possible missing parents. Importantly, in a wider context of pedigree reconstruction (and founder identification), parents/offspring trio detection is desirable since it solves the direction of duos relationships by indicating the ancestors. However, when a complete pedigree is not available, pedigree reconstruction methods based on SD relationships could suggest the direction of a parent/offspring relationship, contributing to build a reliable parentage and to infer information on several sibship clusters.

Even though our panel of accessions was very large, our analyses benefited from the availability of public genotypic datasets developed using a common SNP array (De Lorenzis et al., 2015, 2019; Laucou et al., 2018). By merging all the datasets available, we performed a kinship analyses over a very large number of genotypes (over a thousand), increasing the chance to find new relationships between varieties from different geographic areas. As an example, most of the new relationships of “Visparola” were found with varieties of different datasets, enabling to depict the key international role of this variety. This approach indeed allows to bring to light the often intense and not documented movements of varieties across grape growing regions.

The SNP set we used resulted very powerful for parentage inference, leading to the invalidation of several duos and trios previously obtained using a low number of SSR markers (e.g., Cipriani et al., 2010; Lacombe et al., 2013). Similar findings are reported by Laucou et al. (2018), who conducted a parentage analysis (based on the same SNP array used in our study) and proposed several invalidations of previous SSR-based parentages. The PO relationships between “Moscato bianco” (“Muscat à petits grains blancs”) and “Moscato di Terracina” based on 19 on the 20 investigated loci SSR (Lacombe et al., 2013) is invalidated by both our study and the one by Laucou et al. (2018) SNP-based. About the trio “Carricante” offspring of “Montonico pinto” and “Visparola” proposed by Cipriani et al. (2010), our analysis rejected the first parent in favor of “Strinto porcino” as a genitor.

The software COLONY enables pedigree reconstruction using a likelihood-based method. The main limitation of this approach is related to its high computation demand that restricts its application to relatively small datasets (see, e.g., Crespan et al., 2020; Raimondi et al., 2020). The pedigree reconstruction method adopted in our study had the advantage of being applicable to a very large dataset, since it is not based on computation intensive likelihood estimations. For this reason, SD inference might be less powerful, and findings would need be supported by multiple SD relationships or by additional tests (e.g., counting of Mendelian errors for putative grandparent pairs). This is the case of large SD (half-sibship) clusters, for instance the one including “Caloria”, “Rossara toscana”, “Pollera nera”, all offspring of “Termarina (Sciaccarello)” (see orange ellipses in Supplementary File 9). All those varieties have been found as likely SD of each other, when “Termarina (Sciaccarello)” is tested as parent. Yet, the SD relationship between “Rollo” and “Garganega”, as well as between “Rollo” and “Visparola” are also supported by a very low Mendelian error rate when testing “Garganega” and “Visparola” as “Rollo” grandparents (gray arrows in Supplementary File 9).

Moreover, directions of PO relationships may be supported by an evidence of an FS relationship, such as in the case of “Visparola”. The FS relationship between “Visparola” and “Avgoustiatis” (k0 = 0.23, k1 = 0.42, and k2 = 0.35), together with the fact that “Avgoustiatis” has been found PO with none of the “Visparola” PO relatives, supported “Visparola” as parent for all its PO relationships.

Interestingly, some of the sibship clusters that our study shares with two other previous works SNP-based analyzed by COLONY, show the same relationship pattern. The SD group formed by “Grignolino”, “Arneis”, “Uvalino”, “Neretto di Salto”, “Malvasia aromatica di Parma” and “Nebbiolo” (orange in Supplementary File 9), all varieties also included in the study of Raimondi et al. (2020), is fully compatible with the network proposed by those authors, where a genotype # 2 (reconstructed by COLONY) links all those varieties with the same kin relationships. Looking at “Vulpea” descendants, the two SD groups: “Cianorje” and “Refosco nostrano”, “Glera” and “Pignolo” with “Aghedene”, both comply with the relationships among the same varieties suggested applying COLONY by Crespan et al. (2020).

### The Reconstructed Pedigree of the Main Italian Founders

Several founder varieties of the Italian germplasm emerged from our reconstructed pedigree (red varieties circled in Figure 1; green highlighted varieties in Supplementary File 9).

The varieties originating from controlled crosses and the contributions of foreign varieties were also highlighted in the network (Figure 1). Considering that only a few (21) of the varieties involved were from controlled crosses, most of the analyzed grapes arose from spontaneous hybridization, generally from Italian genitors or from varieties long-cultivated in Italy (“Garganega”, “Sangiovese”, “Mantonico bianco”, etc.). Among the few key genitors considered allochthonous there are “Moscato bianco” and “Achladi”, ancient grapes both of alleged Greek origin. The Italian peninsula should therefore be seen as the place where many traditional grapes were originated.

### Main Genitors From Southern Italy

“Sangiovese” is the most cultivated grapevine variety in Italy: it covers 54,000 ha, which is 7.9% of the total vineyard area (OIV, 2017) although this percentage is decreasing. Its first documented reference is related to Tuscany (Soderini, 1590), where it is now the most important variety. As previously reported (Di Vecchi Staraz et al., 2007; Lacombe et al., 2013; Laucou et al., 2018; De Lorenzis et al., 2019), many “Sangiovese” PO related varieties are traditional in Sicily and Calabria (Southern Italy).

Previously proposed parentages of “Sangiovese” (Vouillamoz et al., 2007; Bergamini et al., 2013) were invalidated by our results. In those pedigrees “Sangiovese” would arise from the crosses of “Ciliegiolo” and “Calabrese di Montenuovo” (a variety from Campania) or “Ciliegiolo” and “Negrodolce” (syn. “Morellino del Valdarno” in Tuscany, according to Crespan et al., 2008), respectively. None of the mentioned two latter grapes, both included in our dataset, are consistent as parents of “Sangiovese”, resulting instead its progeny (Figure 1). “Ciliegiolo”, on the other hand, is confirmed as an offspring of “Sangiovese” and “Moscato violetto (Muscat rouge de Madère)”, as suggested by Di Vecchi Staraz et al. (2007). In our research, the analysis of SD relationships suggested that “Sangiovese” is more likely an offspring of “Strinto porcino” and a SD relative of “Visparola” (Supplementary File 9). However, this pedigree configuration was supported by only one SD relative of “Sangiovese” (i.e., “Visparola”). Therefore, future investigations will be required to confirm it, possibly including both the true parents “Sangiovese”. The crosses reported between “Strinto porcino” and “Visparola” (“Giosana”, “Iusana” and “Carricante”, etc.) might indicate an ancient cultivation of “Strinto porcino”, in agreement with the pedigree we proposed for “Sangiovese”.

“Strinto porcino” has been reported to be a local, very rare variety from Basilicata in Southern Italy (Del Lungo et al., 2016; De Lorenzis et al., 2019), while the white-berried “Visparola” has been described to be cultivated in Sicily by Cupani (1696) and Sestini (1812) as reported by Carimi et al. (2010), although it is currently no longer grown. The four offspring of “Strinto porcino” and “Visparola” suggest that these two varieties were once overlapping their growing area. Among their offspring, “Carricante” is today one of the main white varieties in the wine area around the volcano Etna, in Sicily. Other important offspring of “Sangiovese” are “Frappato”, a well-known black variety from Sicily, and “Gaglioppo”, the important historic wine grape from Calabria. The latter originated from “Sangiovese” and “Mantonico bianco” like the renowned “Nerello mascalese” as also reported by Gasparro et al. (2012).

“Mantonico bianco” is an ancient cultivar attested in Sicily and Calabria since centuries (here documented at least since late XVI century (Marafioti, 1601). “Mantonico bianco” is currently cultivated in the provinces of Reggio Calabria and Crotone, part of the ancient *M. Graecia*. Its numerous offspring are all from Southern Italy: Sicily, Calabria and Basilicata. “Calabrese (Nero d’Avola)”, “Catarratto bianco”, and “Ansonica” are important varieties for the Sicilian wine industry (the latter present also in Tuscany), as well as “Grillo”, issued from a cross between “Catarratto bianco” and “Zibibbo (Moscato d’Alessandria)” as already attested (Di Vecchi Staraz et al., 2007).

**FIGURE 2:**
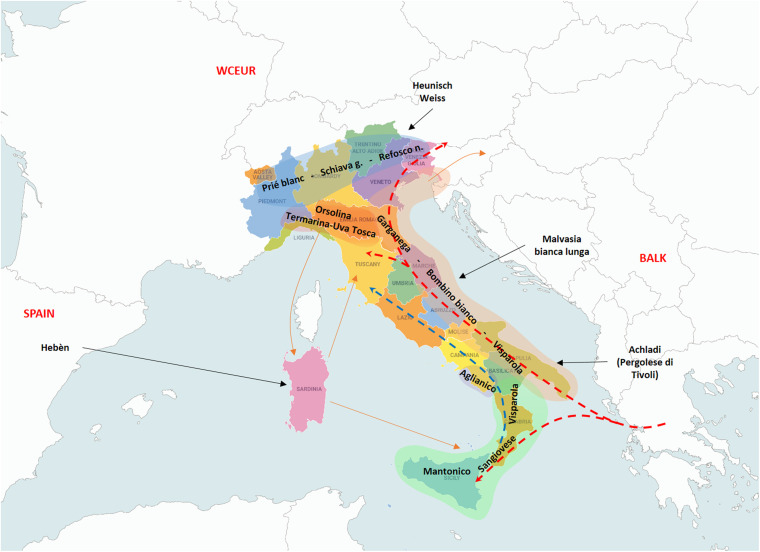
The resulted main founders of the traditional cultivated germplasm are scattered along the Italian peninsula according to their influence geographic areas. Green highlighted area: south-western Italy (IT-SW); Brown area: Italian Adriatic Coast (IT-AC) area of diversification; Red area: north-western Italy (IT-NW) area of diversification; Blue: Italian Northern Borders; Dotted arrows: the direction of the supposed genetic flow related to “Visparola” (red) and “Sangiovese” (blue) along the Italian peninsula, and to “Visparola” in the Balkans; Black arrows: introgression of varieties from outside areas; and Brown arrows: connections among some Italian regions with the Italian main centers of diversification. Map of Italy by Vemaps.com.

Coming to the second degree relationships and their relevance, the main founders “Mantonico bianco”, “Strinto porcino” and even “Visparola” are connected with many varieties from Southern Italy (“Verdone nero”, “Carricante”, “Prunesta (false)”, “Trebbiano antico” and “Lucignola”, etc.) and also with a small cluster of nine grapes from Cilento (Southern Campania), all POs of “Aglianico” (Supplementary File 9). These relationships, together with the already mentioned PO relationships, suggest the kin-groups of “Strinto porcino” and its offspring “Sangiovese”, “Mantonico bianco” and “Aglianico” were present in the same area of South-Western Italy (IT-SW) corresponding to the regions of Sicily, Calabria, Basilicata and Southern Campania (Figure 2).

As “Aglianico” concerns (as well as its offspring), the alleged SD relationships reported by De Lorenzis et al. (2012) with “Syrah” and the Syrah’s parent “Dureza” (claimed avuncular and half-sibling of “Aglianico”, respectively) are not supported by our findings, where “Aglianico” resulted genetically distant from the other main genitors and kin groups.

### The Key Role of “Visparola”

A relevant result of our study was the identification of “Visparola” as a key ancestor of several Italian varieties of Southern and Central Italy (Supplementary File 9). Interestingly, our analysis suggested that “Visparola” would also be the parent of the two important Balkan genitors “Vulpea” and “Alba imputotato”, invalidating the SSR-based parentages previously proposed for these two varieties (Lacombe et al., 2013).

“Visparola” most probably corresponds to the accession “Arvina di Petralia” reported by Lacombe et al. (2013), considering the consistency of five of its POs: “Minutolo”, “Erbisedda” (Carcajolo blanc), “Cannella nera”, “Vulpea” and “Alba imputotato”. This is also supported by the synonymy between “Visparola bianca” and “Petralia Sottana” emerged from the analysis of grapevine accessions in the collection of Baron Antonio Mendola in Sicily by Rovasenda (1877). Recent SSR profile comparison analysis indicates that “Visparola” is also a synonym of “Crepolino” and “Cascarello” from Tuscany (Crespan, unpublished data), and “Rossola (Tebano)” and “Scacco” from Emilia Romagna (Pastore et al., 2020).

“Vulpea”, one of the varieties in this study indicated as “Visparola’s” progeny, is in turn a prolific genitor, having given rise to many offspring, in Italy (Crespan et al., 2020 and this study) and in the Balkan peninsula (Lacombe et al., 2013; Žulj Miahljeviæ et al., 2020). Both these works suggested a pedigree for “Vulpea” (syn. “Blank blauer” and “Bljuzgava”, respectively) where “Visparola” is not involved. “Vulpea” in North-Eastern Italy turned to be hidden under the names of several minor local varieties (“Doretta”, “Quaiara”, “Rosetta” and “Schiavetta”, as reported by Crespan et al., 2020). Interestingly, “Vulpea” was found to be a PO with several North-Eastern Italian varieties, such as “Boschera” and “Molinara” (found in this study), “Glera” (the principal variety of Prosecco wine, which appeared to be SD related to “Pignolo” and “Verduzzo trevigiano”), “Cjanorie” (SD related to “Refosco nostrano”), and is known to be a genitor of “Bakator kek” and “Pecsi dinka” from Hungary (Lacombe et al., 2013).

As for “Alba imputotato”, another Balkan variety PO linked to many grapes from that area, our findings rejected the previously proposed pedigree (“Sarfeher” X “Hamvas” by Lacombe et al., 2013) in favor of “Visparola” as genitor. “Alba imputotato” likewise “Vulpea”, is present in Italy (Emilia Romagna) since 1,800 under the local name of “Bisetta” (Imazio et al., 2015).

The area of origin of “Visparola”, as well as its prolific offspring “Vulpea” and “Alba imputotato” remains questionable. The full-sibship between “Visparola” and “Avgoustiatis”, a typical variety from Greece, would suggest a Greek origin for “Visparola”. Moreover, using the IBD kinship coefficients we found “Verdeca” (“Lagorthi” in Greece), an offspring of “Malvasia bianca lunga”, as close relative of “Visparola” (k = 0.18) and “Avgoustiatis”. The alleged Moldovan “Coarna alba” appeared related (k = 0.14) to “Visparola” as well (Supplementary File 9). All these findings seem to indicate a Balkan origin for “Visparola” and/or its widely ancient spreading across the Central-Eastern Mediterranean area.

“Maiolica”, another prolific offspring of “Visparola”, has been registered in Marche and Abruzzo (on the Adriatic coast), and more recently in Tuscany as “Sanforte”. Most of its PO related varieties (and their progeny) are from Southern Italy (Apulia, Basilicata, and Campania). In addition, the “Maiolica” shared an offspring with one of the main genitors from the IT-AC, the “Bombino bianco”, had connections with other varieties from Central Italy (“Bellone” and “Pampanaro”) through “Arsilico”, and “Negro amaro”, one of its offspring, crossed with “Malvasia bianca lunga” and a descendant of “Heunisch Weiss”. Indeed “Maiolica” seems indicate the spreading of “Visparola” along the Eastern Italian Cost.

Following the distribution of Visparola’s synonyms and descendants, a route can be traced in Italy from the South (Sicily, Calabria, and Apulia), along the peninsula to the North-East, toward the Balkan area. This assumption is seductive as it would match the westward expansion of viticulture from Greece to Southern Italy due to the Greek domination, and from the South toward central Europe and Northern Balkan with Romans. However, an alternative or simultaneous Balkan route for “Visparola” movement cannot be excluded, especially considering its Balkan relatives. The alleged “Vulpea” cradle in Italy is made less likely by its SD relationships with “Battraube” (Supplementary File 9), parent of “Prokupac” and “Urbanitraube”. This would indicate a Balkan origin for the second Vulpea’s parent. A similar assumption on a possible Balkan origin could be deduced also for “Alba imputotato”, due its SD sibship with “Baratcsuha szuerke”, a grape of alleged Hungarian origin.

### Main Genitors From the Italian Adriatic Coast

In addition to “Visparola”, “Garganega”, “Malvasia bianca lunga” and “Bombino bianco” chiefly contributed to variety assortment along the Adriatic costal area of Italy (Figure 2). Moreover, the last two are involved in the pedigree of varieties typical of both Italian and Balkan peninsulas (this study, Lacombe et al., 2013; Žulj Miahljeviæ et al., 2020).

“Malvasia bianca lunga” is one of the most widespread Malvasias in Italy. Its historic presence under the names of “Maraština” and “Pavlos” is attested in the Balkans (Šimon et al., 2007). At the same time, the progeny of “Malvasia bianca lunga” includes varieties (“Vitouska”, “Malvasia di Lecce” and “Malvasia nera di Basilicata”, etc.) distributed on both sides of the Adriatic Sea.

“Bombino bianco” (“Trevolina” and “Puljizanac” in the Balkans), shows a large PO group mainly widespread along the Italian peninsula eastern side. Its kin group includes “Montepulciano”, one of the most important varieties in central Italy. The relatively high IBD kinship (k = 0.16) between “Bombino bianco” and “Garganega”, a main founder likely confined to Italy, would suggest an Italian origin for this variety (Supplementary File 9).

“Garganega”, with its first quotation in 1300 (by de Crescentiis, 1309), is one of the earliest mentioned varieties in Italy. Most of its 12 PO related include varieties found along the IT-AC, but also from other areas of the whole Italian Peninsula, and some successful emigrants like “Trebbiano toscano” alias “Ugni blanc”. Although the direction of the PO “Garganega” and “Trebbiano toscano” could not be unequivocally defined, the identification of “Koevidinka feher” as SD (when “Garganega” is tested as parent) indicated that “Trebbiano toscano” is most probably an offspring of “Garganega”.

“Achladi”, a grape of presumed Greek origin that we identify as the historic “Pergolese di Tivoli”, gave origin also to some Italian varieties. Yet this grape was reported as offspring of “Uva sogra” and “Cacciò di fermo nero” (Cipriani et al., 2010). Our findings rejected this pedigree, proposing instead the mentioned parents as Achladi’s progeny, together with many other traditional varieties, mostly for table grape, common along the IT-AC, but also from France, Spain and Greece. Moreover, the SD relationships analysis revealed a sibship cluster of its descendant “Uva sacra (Uva sogra)”, with the two grapes “Plyto” (an ancient, at present neglected variety of the island of Crete) and “Assyrtiko”, one of the major and most renowned wine grapes in Greece (Supplementary File 9). This would support the Southern Balkan origin for both “Uva sacra (Uva sogra)” and “Achladi (Pergolese di Tivoli)”.

### Main Genitors From Central Italy and North-Western Italy

“Termarina (Sciaccarello)”, “Orsolina” and “Uva Tosca” appeared as founder of three other kin groups (Figure 1) mainly linked to IT-NW Italy (Figure 2).

“Termarina (Sciaccarello)” is another of the main founder of the Italian varieties, with numerous PO relationships already reported (Di Vecchi Staraz et al., 2007). This study revealed some additional PO relatives of “Termarina”, indicating that most of them are its offspring (Figure 1). “Termarina” is a seedless red-skinned accession from Emilia Romagna, where it was quoted as early as the XVII century (Bignami et al., 2015a). “Sciaccarello”, a variety from Corse, is its seeded form, believed by some people to be identical to the ancient grape “Mammolo” from Tuscany. Since the identity “Mammolo”-“Sciaccarello” is questioned by some local experts, we included in our dataset the registered clone “Mammolo” I-MAM-PA-1, that resulted a distinct genotype. This turned out to be PO related to “Termarina”, like other varieties recovered by D’Onofrio et al. (2016) in the Northern part of Tuscany.

One of the Termarina’s offspring, “Moscato violetto” is in partnership with Sangiovese the parent of “Centesimino” and “Ciliegiolo”, the latter attested centuries ago in Tuscany (Soderini, 1590) and both grown on one or on both sides of the Apennine range between Tuscany and Emilia Romagna. The same current area of cultivation and of historic occurrence is given by most of the other members of “Termarina” and “Uva Tosca” kin groups (Figure 1). The mentioned founders, the historic “Fortana” and the SD related “Spergola” (alias “Vernaccia di Oristano”) are all quoted as early as XVII century on the Apennine foothills (D’Onofrio et al., 2015; Bignami et al., 2015a). This would support the Northern Apennines area as the likely homeland for all these grapes, including “Spergola”, later introduced to Sardinia as already asserted on the base of other genetic considerations (Raimondi et al., 2020).

Interestingly, using IBD coefficients to further explore “Termarina (Sciaccarello)” relationships, we found it also closely related (k > 0.13) to “Strinto porcino” and to the varieties of the sibship cluster including “Pecorino”, “Cesanese di Affile”, “Pecorello” and “Vernaccina” (Supplementary File 9): all of them are minor varieties currently cultivated from Central to Southern Italy. The mentioned sibship might indicate Termarina’s geographic connection also with the Central-Southern part of the peninsula.

“Orsolina” was first documented by Filippo Re in 1,800 among the grapevine varieties of the mountains of the Reggio Emilia Apennines (Emilia-Romagna region), and later (1,825) it was described by Acerbi among the varieties of Brescia (Lombardia) and cited in 1915 as “Ova orslina” by Casali (Bignami et al., 2015b). The large kin group of “Orsolina” merges varieties mainly from North and North Western Italy. Under the historic synonym of “Coccalona nera”, used in Piedmont, and “Rohrtraube blaurot”, used in lower Germany, this variety has been already identified as a genitor of many grapevines, together with one of its parent (Raimondi et al., 2020). In the present study, several additional offspring of “Orsolina”-“Coccalona”-“Rohrtraube” were identified; most of them comes from Emilia Romagna and Tuscany, raising the offspring number of this prolific variety to as many as 25. The present study confirmed the pedigree of “Moscato nero d’Acqui” and “Vespolina”, and the sibship “Barbera”-“Riesling italico” (alias “Welsch Riesling”, “Graševina”), both progenies of “Orsolina” and widely cultivated in Northern Italy and in the Balkan countries, respectively. The parentage of many aromatic grapevines typical of North Western Italy previously reported (Ruffa et al., 2016) were also confirmed, with the Moscato bianco’s offspring “Malvasia aromatica di Parma” as main genitor (Figure 1). The latter aromatic cultivar resulted linked by SD relationship with “Koevidinka feher”, an alleged neutral Hungarian variety that our findings do not confirm as offspring of “Papazkarasi” as previously suggested (Lacombe et al., 2013).

### Variety Migrations

The outcomes of this extended parentage analysis allowed us to roughly outline the main lines of the cultivar movement affecting the Italian peninsula. Even though our variety set represented a large part of the Italian germplasm and of the grapes from Europe, Mediterranean and Caucasus area, still many minor varieties were not included. Therefore, some conclusions on variety spreading and varieties origin might be partial.

An important genetic contribution comes to Southern Italy from the Southern Balkan area (mainly Greece), through the possible introduction of founders such as “Visparola” and “Achladi (Pergolese di Tivoli)”. To these two relevant emigrants, “Moscato bianco” and “Zibibbo (Muscat of Alexandria)”, presumably of Greek origin too, must be added. A flow from Balkans to Eastern Italy took likely place as well as in the opposite direction as “Bombino bianco” would suggests.

A hypothetical spreading within the Italian peninsula could be traced with “Visparola” from South to North-East (Figure 2), likely along the Eastern side, and “Sangiovese” from South to Central Italy.

A genetic contribution from foreign areas occurred in the island of Sardinia, where Hebèn, one of the major founder of the Iberian grape assortment, resulted the genitor of the local varieties “Monica”, “Nieddu Mannu” and “Torbato” (Supplementary File 9). As an example of variety movement in the opposite direction, from Italy to the Iberian Peninsula, there is the intriguing migration (followed by local hybridization) of “Priè blanc” (alias “Agostenga”) from Northern Italy to Central Spain (Schneider et al., 2010).

Finally, an alleged contribution from Central Europe to Northern Italy relys on the ubiquitous “Heunisch weiss” (the well known “Gouais blanc”) through the already reported Italian offspring “Ribolla gialla” and “Schiava” (“Rossara”; Maul et al., 2015; Crespan et al., 2020). Furthermore, West-Central European germplasm was intensively used in Italy under recent breeding programs for wine grape varieties (Figure 1).

The kin groups of “Orsolina”, “Termarina” and “Uva tosca” seems to indicate an effect on the varietal assortment related to Central and North-Western part of Italy (Figure 2). Finally, the muscat founders (“Moscato bianco” and “Zibibbo”), spread their genes over the whole Italy, with a high contribution by the former to the North-Western of the peninsula (Figure 1), where aromatic grapevines were intensively cultivated and selected (Ruffa et al., 2016). As donors of the peculiar grape aromatic flavor due to the high terpen content, “Moscato bianco” and “Zibibbo (Moscato d’Alessandria)” deeply contributed to the overall European cultivated germplasm. Due to their climate requirements, the cultivation of “Moscato bianco” and its offspring has moved further North, while the influence of “Zibibbo (Moscato d’Alessandria)” remained limited to the Mediterranean basin.

## Conclusion

A SNP-based genetic atlas of a large part of the Italian grapevine germplasm has been provided for the first time and several conclusions can be drawn from this study.

The first concerns technical considerations about the analytical tools applied. The availability of the PN40024 reference sequence and the re-sequencing data from a panel of 50 Italian grape varieties carried out in the frame of Vigneto project was the starting point for the development of an Infinium 18K Grape Array shared with other studies. This sharing improved common analytical tool, permitted the subsequent merging of data, and therefore provided a greater analytical power to evaluate, among other, relationships existing not only inside the Italian grape germplasm, but even between Italian and foreign grapevines. The subsequent kinship analysis and pedigree reconstruction carried out taking into account first and second degree relationships were effective in grapevine, thanks to the predominant use of vegetative propagation. Additionally, the genotyping data produced can be useful in perspective even to evaluate the genetic structure of Italian grape germplasm and to map, in connection with phenotyping data, viticultural traits, including the qualitative ones, to be exploited in breeding programs.

The second consideration is that the atlas produced a huge amount of genetic information, in terms of relationships among varieties. Several connections were identified for the first time, whereas relationships identified in previous studies were confirmed or denied. Kinship relationships (PO, FS, and SD relationships) clearly indicated the key role of few varieties in the evolution of the cultivated Italian grapevine germplasm.

A panel of main genitors were identified (“Strinto porcino” and its offspring “Sangiovese”, “Visparola”, “Mantonico bianco”, “Aglianico”, “Termarina (Sciaccarello)”, “Bombino bianco”, “Garganega”, “Orsolina”, “Uva tosca”). Some of these varieties seem more closely linked to one of the three Italian geographical and climatic macro-areas (North, Center and South), while others seem to have a general significance for the entire territory. For example, the centrality of “Visparola” in the origin of Italian germplasm is clear.

Pedigree reconstruction, together with the identification of the main ancestors of traditional Italian cultivars and closely-related varieties, is considered of great value also for the purposes of their oenological and viticultural enhancement. In fact, this information has appeal on wine marketing since it is useful for storytelling and for connections with the “terroir”.

Furthermore, the results of this study also provide useful information on the diversity of the grapevine germplasm preserved in some of the largest Italian collections. The genotyping tools developed can increase the efficiency of germplasm management, helping to identify duplicate accessions, solving misnomer issues and filling genetic gaps in the collections.

Finally, this atlas contributes to the description and valorization of the native grapevines, generally those with a longer and more positive history of privileged relationship with terroirs suited to produce quality wines; from a commercial point of view, this is a strong point of the Italian viticultural-enological system, which can offer on the world market many unique and unrepeatable products linked to a territory.

## Data Availability Statement

The raw data supporting the conclusions of this article will be made available by the authors, without undue reservation.

## Author Contributions

CD’O, GT, MG, MC, AS, CB, LP, MB, VN, and VT designed the experiments. CD’O, MG, AS, CB, LP, MB, MM, MC, and VN collected the specimens. CD’O and GT analyzed the data. CD’O, GT, AS, and VT discussed the data and wrote the manuscript. All the authors contributed to the identification of variety names, synonyms, wrong denominations, and misnomers. All the authors contributed to editing the final version of the manuscript.

## Conflict of Interest

The authors declare that the research was conducted in the absence of any commercial or financial relationships that could be construed as a potential conflict of interest.

## Footnotes

1 http://catalogoviti.politicheagricole.it/catalogo.php

## Supplementary Material

Supplementary File 1 In spreadsheet 1: List of the accessions genotyped in this study, their passport data including variety names, berry skin color, grape flavor according to descriptor OIV 236, the varieties as coded by the International *Vitis* Variety Catalogue (VIVC), the Italian *Vitis* Database (IVD) and the Italian National Variety Register (RNVV), respectively, the repositories where the accessions are maintained, the presumed variety countries of origin (according to VIVC); the place where the accessions were collected, and the variety presumed geographic groups (BALK: Balkans; IBER: Iberian Peninsula; ITAP-north, -center, -south: Italian Peninsula north, -center, -south, respectively; EMCA: Eastern Mediterranean and Caucasus; MFEAS: Middle and Far East; NEWO: New World; and WCEUR: Western-Central Europe. In spreadsheet 2: List of the analyzed non-redundant varieties: genotyped in this study **(A)**, with not matching genetic profiles added from literature **(B)**. Variety names was assigned according to Vitis databases or identify the variety on the base of its genetic relationships. Passport data are shown as in spreadsheet 1; synonyms, misnomers and wrong denominations among the varieties of this study and the varieties from literature are also shown. Wrong denominations: incorrect local names of known varieties, here marked with “(false)”. Misnomers: errors in grapevine collections, marked with “(misnomer)”.

Supplementary File 2 Heterozygosity and missingness per individual.

Supplementary File 3 6,770 high-quality SNP profiles of 408 grapevine varieties genotyped at 18 K loci. Original dataset was filtered based on SNP call quality: missing rate per genotype <1% and minor allele frequency (MAF) >5%.

Supplementary File 4 Parent-offspring pair identification: distribution of Mendelian errors and IBD coefficients (k0 and k1). Relationships with k0 < 0.03 (vertical dashed line) were considered PO. The red crosses indicate the expected k0 and k1 values for different types of relationship (PO, k0 = 0, k1 = 1; FS, k0 = 0.25, k1 = 0.5; and HS, k0 = 0.5, k1 = 0.5).

Supplementary File 5 List of varieties with parent-offspring (PO) relationships and relative dataset (spreadsheet 1); list of varieties without direct PO relationships (spreadsheet 2); PO pairs (spreadsheet 3); and related literature (spreadsheet 4). (false): wrong denominations (incorrect local names).

Supplementary File 6 Mendelian error rate for detection of parent-offspring trios.

Supplementary File 7 List of trios offspring/parent 1/parent 2 (spreadsheet 1) and related literature (spreadsheet 2).

Supplementary File 8 Network of parent-offspring (PO) relationships among Italian varieties and main connections with foreign varieties. Presumed variety geographic groups: green: cultivars from the Italian Peninsula (ITA); purple: cultivars from Western and Central Europe (WCEUR); yellow: cultivars from Iberian Peninsula (IBER); red: cultivars from Balkan region (BALK) and also from Western regions; brown: cultivars from Eastern Mediterranean and Caucasus (EMCA); blue: cultivars from New World group (NEWO), light blue: cultivars from Magreb (MAGH); and colorless circled black: unknown geographic origin.

Supplementary File 9 The reconstructed pedigree of Italian varieties, including full-sib (FS) and second-degree relationships (SD). Blue solid line: parent-offspring relationship (with arrow when direction is known or deduced); Black square: group of offspring of the same variety; Gray dashed line: SD relationship found using the method of van de Weg et al. (2018); Red dashed line: SD relationship based on IBD coefficients; Yellow ellipse: group of varieties in SD relationships (i.e., putative sibship clusters) identified by the method of van de Weg et al. (2018); Gray solid arrow: grandparent-grandchild relationship, based on the rate of Mendelian errors; Blue dashed line: full-sib relationship. Italian founders were highlighted in green.

## Abbreviations

FS: full-sibs
IBD: identity by descent
LOD: logarithm of the odds
Offs: offspring
PO: parent-offspring
SD: second-degree relationship
SNPs: single nucleotide polymorphisms.
