# Supplementary figures and images for: Parentage Atlas of Italian Grapevine Varieties as Inferred From SNP Genotyping
Source: Front Plant Sci. 2021 Jan 15;11:605934. doi: 10.3389/fpls.2020.605934 (PMC7874015; doi:10.3389/fpls.2020.605934)

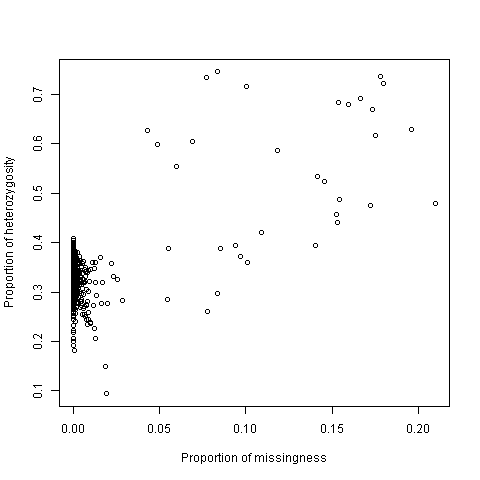

Supplement: Supplementary File 1 — In spreadsheet 1: List of the accessions genotyped in this study, their passport data including variety names, berry skin color, grape flavor according to descriptor OIV 236, the varieties as coded by the International Vitis Variety Catalogue (VIVC), the Italian Vitis Database (IVD) and the Italian National Variety Register (RNVV), respectively, the repositories where the accessions are maintained, the presumed variety countries of origin (according to VIVC); the place where the accessions were collected, and the variety presumed geographic groups (BALK: Balkans; IBER: Iberian Peninsula; ITAP-north, -center, -south: Italian Peninsula north, -center, -south, respectively; EMCA: Eastern Mediterranean and Caucasus; MFEAS: Middle and Far East; NEWO: New World; and WCEUR: Western-Central Europe. In spreadsheet 2: List of the analyzed non-redundant varieties: genotyped in this study (A), with not matching genetic profiles added from literature (B). Variety names was assigned according to Vitis databases or identify the variety on the base of its genetic relationships. Passport data are shown as in spreadsheet 1; synonyms, misnomers and wrong denominations among the varieties of this study and the varieties from literature are also shown. Wrong denominations: incorrect local names of known varieties, here marked with “(false)”. Misnomers: errors in grapevine collections, marked with “(misnomer)”. [file Data_Sheet_1.zip › SupplementaryFile3.png]

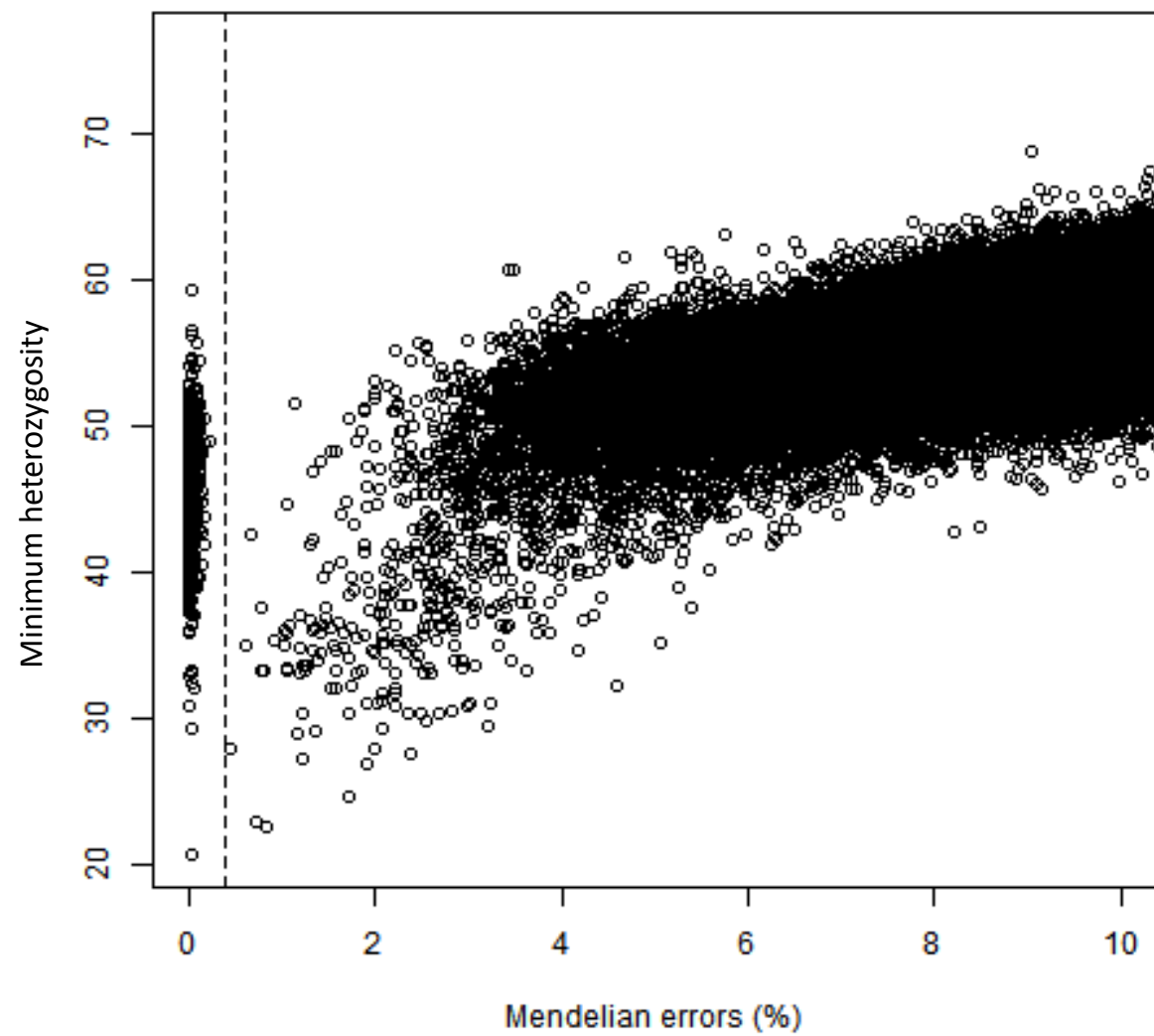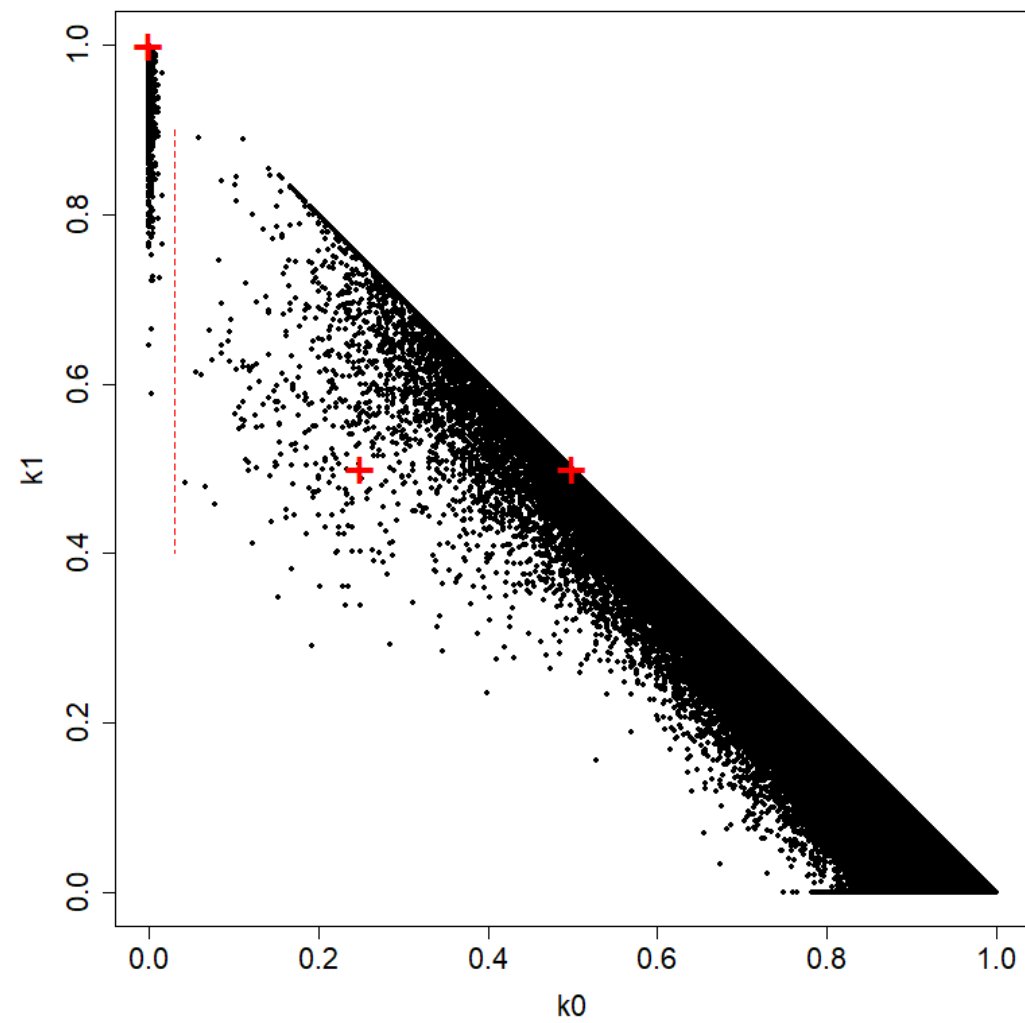

Supplement: Supplementary File 1 — In spreadsheet 1: List of the accessions genotyped in this study, their passport data including variety names, berry skin color, grape flavor according to descriptor OIV 236, the varieties as coded by the International Vitis Variety Catalogue (VIVC), the Italian Vitis Database (IVD) and the Italian National Variety Register (RNVV), respectively, the repositories where the accessions are maintained, the presumed variety countries of origin (according to VIVC); the place where the accessions were collected, and the variety presumed geographic groups (BALK: Balkans; IBER: Iberian Peninsula; ITAP-north, -center, -south: Italian Peninsula north, -center, -south, respectively; EMCA: Eastern Mediterranean and Caucasus; MFEAS: Middle and Far East; NEWO: New World; and WCEUR: Western-Central Europe. In spreadsheet 2: List of the analyzed non-redundant varieties: genotyped in this study (A), with not matching genetic profiles added from literature (B). Variety names was assigned according to Vitis databases or identify the variety on the base of its genetic relationships. Passport data are shown as in spreadsheet 1; synonyms, misnomers and wrong denominations among the varieties of this study and the varieties from literature are also shown. Wrong denominations: incorrect local names of known varieties, here marked with “(false)”. Misnomers: errors in grapevine collections, marked with “(misnomer)”. [file Data_Sheet_1.zip › SupplementaryFile4.pdf]

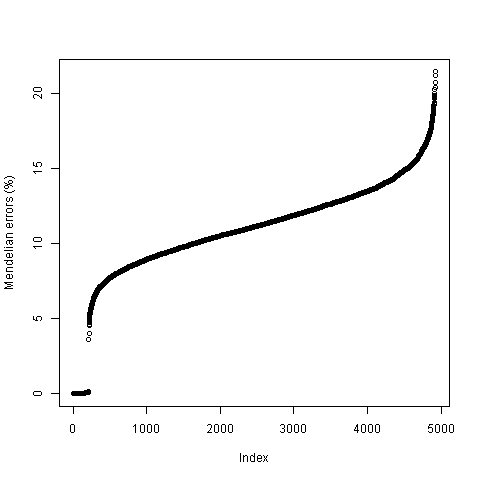

Supplement: Supplementary File 1 — In spreadsheet 1: List of the accessions genotyped in this study, their passport data including variety names, berry skin color, grape flavor according to descriptor OIV 236, the varieties as coded by the International Vitis Variety Catalogue (VIVC), the Italian Vitis Database (IVD) and the Italian National Variety Register (RNVV), respectively, the repositories where the accessions are maintained, the presumed variety countries of origin (according to VIVC); the place where the accessions were collected, and the variety presumed geographic groups (BALK: Balkans; IBER: Iberian Peninsula; ITAP-north, -center, -south: Italian Peninsula north, -center, -south, respectively; EMCA: Eastern Mediterranean and Caucasus; MFEAS: Middle and Far East; NEWO: New World; and WCEUR: Western-Central Europe. In spreadsheet 2: List of the analyzed non-redundant varieties: genotyped in this study (A), with not matching genetic profiles added from literature (B). Variety names was assigned according to Vitis databases or identify the variety on the base of its genetic relationships. Passport data are shown as in spreadsheet 1; synonyms, misnomers and wrong denominations among the varieties of this study and the varieties from literature are also shown. Wrong denominations: incorrect local names of known varieties, here marked with “(false)”. Misnomers: errors in grapevine collections, marked with “(misnomer)”. [file Data_Sheet_1.zip › SupplementaryFile6.png]

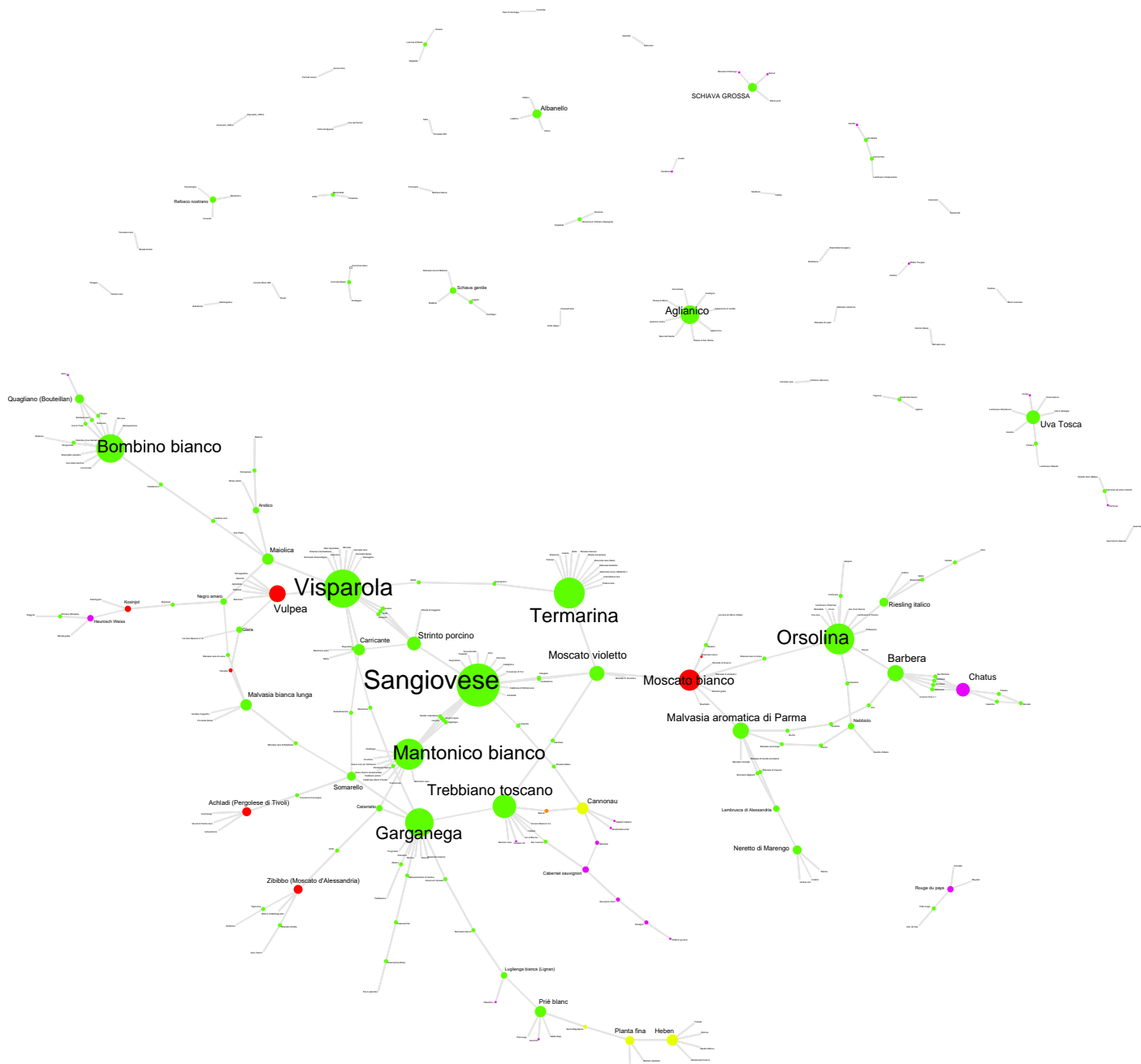

Supplement: Supplementary File 1 — In spreadsheet 1: List of the accessions genotyped in this study, their passport data including variety names, berry skin color, grape flavor according to descriptor OIV 236, the varieties as coded by the International Vitis Variety Catalogue (VIVC), the Italian Vitis Database (IVD) and the Italian National Variety Register (RNVV), respectively, the repositories where the accessions are maintained, the presumed variety countries of origin (according to VIVC); the place where the accessions were collected, and the variety presumed geographic groups (BALK: Balkans; IBER: Iberian Peninsula; ITAP-north, -center, -south: Italian Peninsula north, -center, -south, respectively; EMCA: Eastern Mediterranean and Caucasus; MFEAS: Middle and Far East; NEWO: New World; and WCEUR: Western-Central Europe. In spreadsheet 2: List of the analyzed non-redundant varieties: genotyped in this study (A), with not matching genetic profiles added from literature (B). Variety names was assigned according to Vitis databases or identify the variety on the base of its genetic relationships. Passport data are shown as in spreadsheet 1; synonyms, misnomers and wrong denominations among the varieties of this study and the varieties from literature are also shown. Wrong denominations: incorrect local names of known varieties, here marked with “(false)”. Misnomers: errors in grapevine collections, marked with “(misnomer)”. [file Data_Sheet_1.zip › SupplementaryFile8.pdf]

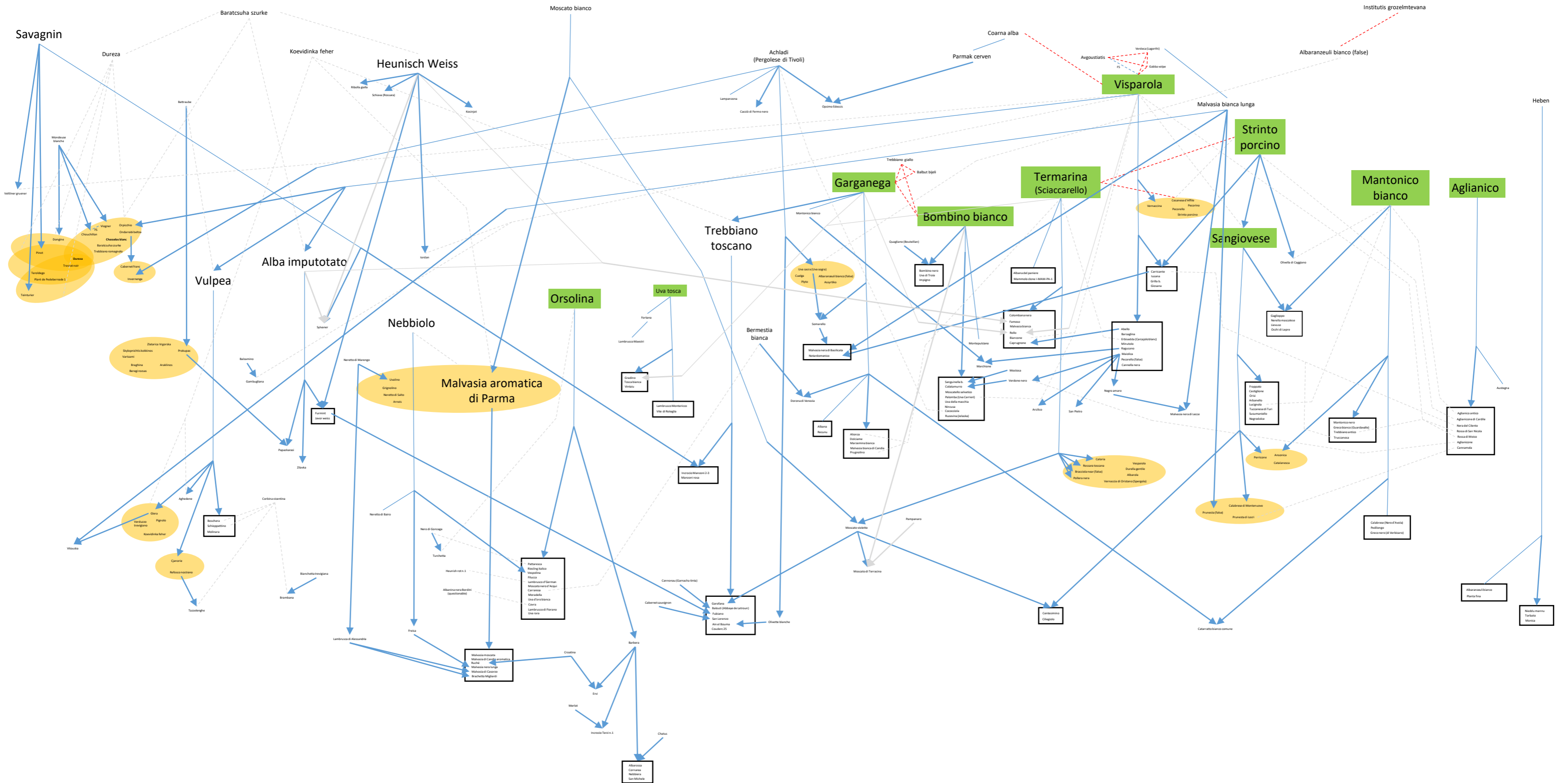

Supplement: Supplementary File 1 — In spreadsheet 1: List of the accessions genotyped in this study, their passport data including variety names, berry skin color, grape flavor according to descriptor OIV 236, the varieties as coded by the International Vitis Variety Catalogue (VIVC), the Italian Vitis Database (IVD) and the Italian National Variety Register (RNVV), respectively, the repositories where the accessions are maintained, the presumed variety countries of origin (according to VIVC); the place where the accessions were collected, and the variety presumed geographic groups (BALK: Balkans; IBER: Iberian Peninsula; ITAP-north, -center, -south: Italian Peninsula north, -center, -south, respectively; EMCA: Eastern Mediterranean and Caucasus; MFEAS: Middle and Far East; NEWO: New World; and WCEUR: Western-Central Europe. In spreadsheet 2: List of the analyzed non-redundant varieties: genotyped in this study (A), with not matching genetic profiles added from literature (B). Variety names was assigned according to Vitis databases or identify the variety on the base of its genetic relationships. Passport data are shown as in spreadsheet 1; synonyms, misnomers and wrong denominations among the varieties of this study and the varieties from literature are also shown. Wrong denominations: incorrect local names of known varieties, here marked with “(false)”. Misnomers: errors in grapevine collections, marked with “(misnomer)”. [file Data_Sheet_1.zip › SupplementaryFile9.pdf]
